# Baseline histological and morphometric description of the retina in the Pied Crow (*Corvus albus*)

**DOI:** 10.1186/s13104-026-07762-1

**Published:** 2026-03-11

**Authors:** Olanrewaju I. Fatola, Taidinda T. Gilbert, Ifukibot L. Usende, James O. Olopade

**Affiliations:** 1 Department of Veterinary Anatomy, Faculty of Veterinary Medicine, University of Ibadan, Ibadan, Nigeria; 2 Sidero Bioscience, Hummelstown, PA 17036 USA; 3 Department of Veterinary Anatomy, Faculty of Veterinary Medicine, University of Abuja, Abuja, Nigeria

**Keywords:** Avian vision, *Corvus albus*, NeuN immunohistochemistry, Photoreceptors, Pied Crow, Retina

## Abstract

**Objective:**

The Pied Crow (*Corvus albus*) is a visually guided diurnal corvid widely distributed across sub-Saharan Africa, yet detailed descriptions of its ocular microanatomy remain limited. This study provides a concise histological and immunohistochemical characterization of the retina from an adult male specimen obtained through an ethically approved wildlife surveillance programme. Hematoxylin and eosin staining, Alcian Blue staining, and NeuN immunolabeling were used to document retinal organization and neuronal localization. The objective was to generate baseline morphological data for this species.

**Results:**

The retina exhibited a well-organized multilayered structure typical of diurnal avian species, supported by a prominent hyaline scleral cartilage plate. The outer nuclear layer contained 3–4 rows of nuclei, consistent with the duplex organization of avian retinas. Total retinal thickness measured approximately 303 μm, with the ganglion cell layer measuring ~ 27 μm. NeuN immunolabeling showed strong nuclear immunoreactivity in the ganglion cell layer and moderate labeling in the inner nuclear layer. NeuN-positive neuronal somata in the ganglion cell layer ranged from 2.4 to 5.8 μm in diameter. These findings provide baseline histological and morphometric reference data for *Corvus albus*, contributing foundational information for future comparative and functional investigations.

## Introduction

The Pied Crow (*Corvus albus*) is a diurnal corvid widely distributed across sub-Saharan Africa, including southwestern Nigeria, where it inhabits both natural ecosystems and human-modified environments [1]. Members of the family Corvidae are recognized for advanced cognition and a strong reliance on visually guided behaviours such as foraging, navigation, and complex social interactions [2–4]. Despite the ecological prominence and behavioural sophistication of *C. albus*, detailed anatomical information on its ocular structures, particularly retinal organization, remains limited.

The eye specimen examined in this study originated from an ethically approved wildlife surveillance programme conducted under the Humboldt Research Hub for Zoonotic Arbovirus Diseases (HRH-ZAD), University of Ibadan. As part of routine post-mortem tissue collection from sampled wildlife, both eyes from a single adult male Pied Crow were obtained for histomorphological assessment. This work extends our broader investigations into the ocular anatomy of African wildlife species [5], including recent histological studies on tree squirrel eyes [6, 7] and related comparative efforts [8] (in press).

This Research Note reports observations from a single opportunistically obtained specimen and does not aim to assess inter-individual variability. The objective is to provide a concise descriptive account of the retinal microarchitecture of *C. albus*, encompassing laminar organization, neuronal localization, and photoreceptor layer characteristics, together with limited morphometric observations to provide reference-scale measurements. Using routine hematoxylin and eosin staining, Alcian Blue staining, and NeuN immunolabeling, the study documents baseline structural features relevant to diurnal visual organization and establishes a reference framework for future comparative and region-specific analyses of African corvid visual systems.

## Main text

### Methods

One adult Pied Crow (*Corvus albus*) was humanely captured in southwestern Nigeria. The specimen was obtained as part of an ongoing wildlife surveillance programme conducted under the Humboldt Research Hub for Zoonotic Arbovirus Diseases (HRH-ZAD), University of Ibadan. Capture and scientific use were carried out in accordance with applicable national and regional wildlife-use regulations for non-protected species and were authorized through institutional ethical approval granted by the Animal Care Committee of the National Veterinary Research Institute, Vom, Nigeria (Approval No. NVRI/AEC/03/11622). Field activities complied with Nigerian wildlife regulations under the Federal Department of Forestry and relevant state environmental authorities, under which non-protected species used in institutional research surveillance do not require individual collection permits. Within this framework, institutional animal ethics approval constitutes the governing authorization for the capture and scientific use of such specimens.

The bird was housed briefly (1–2 h) prior to euthanasia to minimize handling stress. Deep anaesthesia was induced using intramuscular xylazine (10 mg/kg) and ketamine (100 mg/kg). Euthanasia was achieved by anaesthetic overdose, and death was confirmed by cessation of cardiac and respiratory activity before further procedures. Transcardial perfusion was then performed using 10% neutral buffered formalin (NBF). Both eyes were enucleated and post-fixed in 10% NBF for 24–48 h.

Tissues were processed using a Leica ASP 300 S automated tissue processor, dehydrated through graded ethanol, cleared in xylene, infiltrated with paraffin, and embedded in paraffin blocks. Serial Sects.  (2–4 μm) were cut using a Microm HM340E rotary microtome and mounted on glass slides. Hematoxylin and eosin (H&E) staining was used to evaluate general retinal morphology, while Alcian Blue with Kernechtrot counterstain was applied to assess scleral cartilage.

Immunohistochemical detection of neuronal nuclei was performed using a monoclonal mouse anti-NeuN antibody (Chemicon, MAB377; 1:200). Heat-induced epitope retrieval was conducted in 10 mM citrate buffer (pH 6.0). Signal amplification was achieved using the avidin–biotin–peroxidase method (Vectastain PK-6100), and immunoreactivity was visualized with 3,3′-diaminobenzidine (DAB), followed by hematoxylin counterstaining. NeuN labeling was applied to confirm neuronal localization within retinal layers and was not used for subtype-specific identification.

Slides were examined using a Zeiss Axio Imager.A1 microscope equipped with 20× and 40× objectives and a Zeiss IC 3 digital camera. Descriptive morphometric measurements were obtained from representative fields using calibrated digital image analysis (Motic Images Plus 2.0). Measurements included total retinal thickness, individual layer thicknesses, NeuN-positive cell counts, and soma diameters. These values are presented as descriptive observations only.

## Results and discussion

**Fig. 1:**
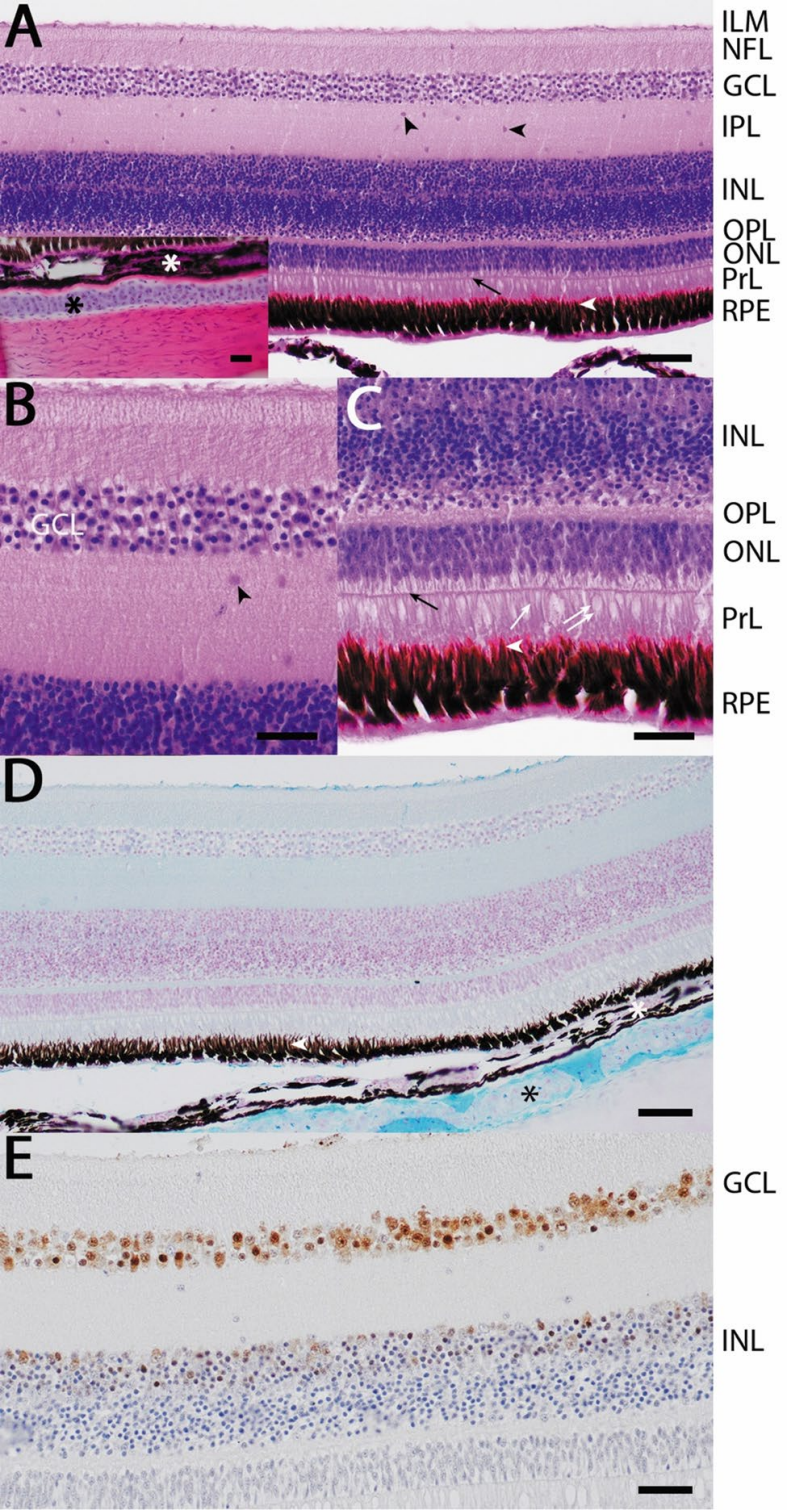
Retinal microarchitecture of the Pied Crow (Corvus albus).**A** H&E staining shows a densely pigmented and vascular choroid (white asterisk) underlying the multilayered avascular retina. Distinct retinal layers are identifiable, including the retinal pigment epithelium (RPE); photoreceptor layer (PrL); outer nuclear layer (ONL) with multiple rows of nuclei and a clearly defined outer limiting membrane (arrow); outer plexiform layer (OPL); inner nuclear layer (INL); inner plexiform layer (IPL) containing scattered nuclei likely corresponding to displaced neurons (black arrowheads); ganglion cell layer (GCL); and nerve fiber layer with inner limiting membrane (NFL, ILM). The sclera consists of an inner hyaline cartilage plate (black asterisk) and an outer fibrocollagenous layer (inset).** B** Higher-magnification view of the GCL demonstrates a morphologically heterogeneous population of ganglion cell nuclei of varying size and staining intensity.** C** Higher-magnification view of the PrL showing photoreceptors with morphologies consistent with rods (single white arrow) and cones (double white arrow). The inner segments of cone photoreceptors appear vacuolated adjacent to the outer limiting membrane. Apical processes of the RPE (white arrowhead) appear to interdigitate with photoreceptor outer segments.** D** Alcian Blue staining highlights moderate to strong mucopolysaccharide content within the scleral hyaline cartilage.** E** NeuN immunolabeling shows strong neuronal nuclear immunoreactivity in the GCL and mild to moderate positivity within the inner portion of the INL. Scale bars:** A**,** D** = 50 μm;** B**,** C**,** E** and inset = 20 μm.

Histological examination revealed a well-organized, multilayered retina characteristic of diurnal avian species (Fig. 1A). The choroid was densely pigmented and vascular, supporting an avascular retina with clearly demarcated layers. The retinal pigment epithelium (RPE) formed a continuous pigmented band with apical processes interdigitating with photoreceptor outer segments. The outer nuclear layer (ONL) consisted of 3–4 rows of nuclei, consistent with the duplex organization of avian retinas containing both rods and cones [9] (Fig. 1A, C).

Descriptive morphometry indicated a total retinal thickness of approximately 303 μm. Layer thicknesses were as follows: ONL ~ 25 μm, inner nuclear layer (INL) ~ 80 μm, inner plexiform layer (IPL) ~ 58 μm, and ganglion cell layer (GCL) ~ 27 μm. Although derived from a single specimen, these measurements establish an initial anatomical reference range for the species. These values thus provide reference-scale anatomical context but are not intended for statistical inference.

A prominent hyaline cartilage plate was present within the sclera, consistent with typical avian ocular architecture [10, 11]. Alcian Blue staining confirmed the mucopolysaccharide-rich extracellular matrix within this cartilage (Fig. 1D).

The INL exhibited stratification consistent with the organization of bipolar, amacrine, and Müller cell nuclei in vertebrate retinas. Occasional nuclei within the IPL may correspond to displaced ganglion or amacrine cells [12, 13], although definitive identification cannot be made based on morphology alone. Mild vacuolation within photoreceptor inner segments was observed adjacent to the outer limiting membrane. While such vacuolation may relate to cone-associated oil droplets described in diurnal birds [14–17], fixation-related artefact cannot be excluded.

NeuN immunolabeling demonstrated strong nuclear immunoreactivity within the ganglion cell layer (GCL) and mild to moderate labeling in the inner portion of the INL (Fig. 1E). Higher-magnification examination of the GCL (Fig. 1B) revealed a morphologically heterogeneous population of ganglion cell nuclei, varying in size and staining intensity. Within sampled fields, approximately 25 NeuN-positive nuclei per 2000 μm² were observed in the GCL. Somatic diameters of NeuN-positive neurons (approximately 20 cells measured) ranged from 2.4 to 5.8 μm, reflecting variability consistent with mixed ganglion cell populations described in other avian retinas. These measurements are presented as descriptive reference values only, given the single-specimen design.

Overall, the retina of *Corvus albus* exhibits laminar organization, neuronal localization, and scleral structural features consistent with diurnal avian visual systems. While most findings align with established avian retinal characteristics, the present observations provide the first documented histological dataset for this species and establish a baseline reference for future comparative, regional, and quantitative investigations of corvid visual systems.

## Limitations

This study reports baseline retinal histology from a single opportunistically obtained adult male specimen. The single-specimen design precludes assessment of inter-individual variability and limits generalization across age groups, sexes, or ecological populations.

Only one neuronal marker (NeuN) was employed, and it was used solely to confirm neuronal localization rather than to identify specific retinal subtypes. No cell-type-specific immunohistochemical analyses were performed. Morphometric values are descriptive and were not subjected to statistical comparison. Regional retinal comparisons (e.g., central versus peripheral retina or potential foveal specializations) were not conducted. Interpretation of photoreceptor inner-segment vacuolation remains tentative due to possible fixation artefact. No additional tissue was available to permit extended analyses.

These constraints reflect the intentionally descriptive scope of this Research Note and highlight the need for future investigations incorporating larger sample sizes, regional retinal mapping, additional molecular markers, and quantitative methodologies.

## Data Availability

All data generated or analysed during this study are included in this published article and its figures. Additional raw histological or immunohistochemical images are available from the corresponding author on reasonable request.

## Abbreviations

HRH-ZAD: Humboldt Research Hub for Zoonotic Arbovirus Diseases
NBF: Neutral buffered formalin
RPE: Retinal pigment epithelium
ONL: Outer nuclear layer
OPL: Outer plexiform layer
PrL: Photoreceptor layer
ILM: Inner limiting membrane
INL: Inner nuclear layer
IPL: Inner plexiform layer
GCL: Ganglion cell layer
NeuN: Neuronal nuclei antigen
H&E: Hematoxylin and Eosin
DAB: 3,3′-diaminobenzidine
